# Predicting female pelvic tilt and lumbar angle using machine learning in case of urinary incontinence and sexual dysfunction

**DOI:** 10.1038/s41598-023-44964-0

**Published:** 2023-10-20

**Authors:** Doaa A. Abdel Hady, Tarek Abd El-Hafeez

**Affiliations:** 1 Department of Physical Therapy for Women’s Health, Faculty of Physiotherapy, Deraya University, EL-Minia, Egypt; 2 Department of Computer Science, Faculty of Science, Minia University, EL-Minia, Egypt; 3 Computer Science Unit, Deraya University, EL-Minia, Egypt

**Keywords:** Body mass index, Physical examination, Diagnosis, Health services, Occupational health, Computer science, Information technology, Statistics

## Abstract

Urinary incontinence (UI) is defined as any uncontrolled urine leakage. Pelvic floor muscles (PFM) appear to be a crucial aspect of trunk and lumbo-pelvic stability, and UI is one indication of pelvic floor dysfunction. The evaluation of pelvic tilt and lumbar angle is critical in assessing the alignment and posture of the spine in the lower back region and pelvis, and both of these variables are directly related to female dysfunction in the pelvic floor. UI affects a significant number of women worldwide and can have a major impact on their quality of life. However, traditional methods of assessing these parameters involve manual measurements, which are time-consuming and prone to variability. The rehabilitation programs for pelvic floor dysfunction (FSD) in physical therapy often focus on pelvic floor muscles (PFMs), while other core muscles are overlooked. Therefore, this study aimed to predict the activity of various core muscles in multiparous women with FSD using multiple scales instead of relying on Ultrasound imaging. Decision tree, SVM, random forest, and AdaBoost models were applied to predict pelvic tilt and lumbar angle using the train set. Performance was evaluated on the test set using MSE, RMSE, MAE, and R^2^. Pelvic tilt prediction achieved R^2^ values > 0.9, with AdaBoost (R^2^ = 0.944) performing best. Lumbar angle prediction performed slightly lower with decision tree achieving the highest R^2^ of 0.976. Developing a machine learning model to predict pelvic tilt and lumbar angle has the potential to revolutionize the assessment and management of this condition, providing faster, more accurate, and more objective assessments than traditional methods.

## Introduction

Urinary incontinence (UI) can be described as an involuntary loss of urine^1^. Although UI is not a life-threatening illness, it has been found to have a negative impact on QoL in terms of psychological, social, and sexual issues, and it is one of the most common manifestations of PFD^2,3^. One potential mechanism is that PFM, among other things, help to maintain spinal and sacral stability^4^ by mechanically stabilizing the spine and pelvis, as well as by adjusting intra-abdominal pressure^5^. As a result, any issue with PFM may have an adverse effect on lumbopelvic stability. The increased incidence of enlarged lumbar curvature, anterior pelvic tilting, altered thoracic curvature, sacral rotation, and altered both lumbar and pelvic mobility in women with UI compared to women without the condition. These findings could confirm the biomechanical link between PFD and low back pain associated with UI^6^. Pelvic tilt and lumbar angle are important parameters that are used to evaluate the posture and alignment of the spine in the lower back region and pelvis. These parameters have been found to be closely related to female pelvic floor dysfunction, which encompasses a range of conditions such as urinary incontinence, fecal incontinence, and sexual dysfunction. Female pelvic floor dysfunction affects a significant proportion of the female population worldwide and can have a significant impact on the quality of life of those affected^7,8^. Traditional methods of assessing pelvic tilt and lumbar angle involve manual measurements and are often time-consuming and prone to inter-observer variability. However, with the recent advancements in machine learning and computer vision, it is now possible to predict these parameters using non-invasive and automated methods^9^.

The first attempt of AI and its application in public health and medicine specialties were begun in the 1960s, with a major focus on diagnosis and treatment^10^. Ted Shortliff of Stanford University and his pioneering MYCIN project are the most well-known early work in the medical AI field. MYCIN is a rule-based expert system with “if-then” rules and certain values. It was recommended to choose antibiotics for various infectious diseases^11–13^. Although MYCIN has not been used clinically, it has been proven to be superior to human infectious disease experts. In 1982, Scholowitz published a textbook on medical artificial intelligence, which contained a collection of research articles on various topics in the field. For physical therapists who have received the functional diagnosis, biomechanics is one of the best assessment tools^14^. Advanced analysis of the range of motion is done with a goniometer, but as technology advances, you can do more than you think. Use this motion analyzer to record EMG activation and muscle relaxation.

In recent years, the use of machine learning, a type of artificial intelligence technology, has grown substantially in the field of disease prediction and stratification, particularly for complex cases involving multiple factors. Machine learning algorithms can analyze multiple variables to identify important combinations that support disease diagnosis and prognosis, as well as detect nonlinear relationships between them. The technology can work with various types of variables and often produce diagnoses with similar or better accuracy than human clinicians based on large data sets. Moreover, machine learning can uncover latent patterns that may elude even the most experienced clinicians^15,16^. While machine learning is a powerful tool for making predictions and stratifications, it differs from traditional approaches in that it is not based on established principles, but rather on patterns and trends in data. This means that biases within the data can impact the accuracy of its predictions, and there may be challenges in reproducing results. Therefore, it is important to exercise caution when applying machine learning to healthcare, and to carefully consider the potential for bias and variability in the data being analyzed^17^. Furthermore, the development of machine learning models typically involves a vast number of explanatory variables, making it challenging to implement them effectively in everyday clinical practice.

There have been recent reports of postoperative changes in pelvic position that can result in angular shifts in acetabular components^11,12^. In a study by Nishihara et al^13^, the tilting angle of the pelvis following total hip arthroplasty (THA) was measured using a 3-dimensional computed tomography (CT) model and anteroposterior radiograph matching. The researchers defined this tilting angle as the pelvic flexion angle (PFA). The study also found that PFA measurements in different positions, such as sitting, standing, and supine, can affect the angle of the acetabular cup.

Lembeck et al.^12^ reported that cup anteversion changes by 0.7_ per 1_ change in PFA, and Babisch et al.^18^ reported that cup inclination changes by approximately 0.3_ and cup anteversion by approximately 0.8_ per 1_ change in PFA. They reported that 8% of patients who underwent primary THA showed pelvic tilt (PT) more than 20_ posteriorly at 5 years after surgery^11^. In such cases, cup anteversion may change by more than 14, which cannot be ignored. A postoperative change in PFA might be a cause of dislocation due to changes in the functional anteversion of the acetabular component and several reports have indicated that it is important to plan cup placement considering the position of pelvis^19^. Changes in PFA angle after THA may be due to a complex set of confounding factors, that is, age, gender, and spinal mal-alignment due to degenerative changes in intervertebral discs^11^, and it is difficult to predict these changes with conventional statistical methods.

Schwartz et al.^20^ introduce an algorithm to measure lumbopelvic parameters on lateral lumbar radiographs with comparable accuracy to surgeons. The algorithm could be used to streamline clinical workflow or perform large scale studies of lumbopelvic parameters.

### Problem statement

Incontinence and sexual dysfunction are both complex health issues that require a comprehensive understanding of the underlying anatomical and physiological factors that contribute to them. One of these factors is pelvic tilt and lumbar angle, which can be affected by a variety of factors, such as aging, pregnancy, pelvic floor dysfunction and certain medical conditions. However, accurately predicting these changes using traditional diagnostic methods can be challenging and time-consuming. Machine learning algorithms can provide a more efficient and accurate means of predicting changes in pelvic tilt and lumbar angle in cases of incontinence and sexual dysfunction.

### Objectives

The primary objective of this paper is to develop a machine learning algorithm that can accurately predict changes in pelvic tilt and lumbar angle in cases of incontinence and sexual dysfunction. The specific objectives of this project are as follows:

- Gather a large dataset of pelvic tilt and lumbar angle measurements for women with incontinence and sexual dysfunction.
- Determine which machine learning algorithms are most appropriate for predicting pelvic tilt and lumbar angle changes.
- Use feature selection techniques to identify which variables are most important for predicting pelvic tilt and lumbar angle.
- Train and test the machine learning models using the gathered data and cross-validation techniques.
- Interpret the results to determine the accuracy of the models and their ability to predict pelvic tilt and lumbar angle changes.
- Develop a user-friendly interface for healthcare professionals to use the machine learning models to diagnose and treat patients with incontinence and sexual dysfunction.

## Materials and methods

### Trial Design

This cross-sectional study was approved by the Ethical Committee at Deraya University in El-Minya, Egypt (No: 1/2023). The trial followed human research ethics, and all patients supplied written consent after getting a thorough explanation of the investigation. The research was carried performed at an outpatient clinic between February 1 and April 1, 2023. This study’s clinical trial identifier is NCT/05,803,512.

### The sample size

All ultrasound measures were performed by a qualified therapist with 5 years of diagnostic and ultrasonography experience, ensuring consistency. Regarding representativeness, our dataset included over 92 patients spanning various ages, clinical presentations and demographic factors. To eliminate type 2 error, sample size was calculated before to the start of the study. The preceding sample size was estimated using G * Power (Wilcoxon-Mann–Whitney test)^21^, using the statistical indices d = 0.5, with an effect size dz of 0.5, (1−B) = 0.95 power analysis, and a 5% significant threshold on both sides. Based on a recent cross-sectional study studying the relationship between spinal curvature and pelvic organ prolapse, the total estimated sample size was at least 30 women with UI and included fall, with 46 women in each group.

### Eligibility criteria

All of the women included in the study were initially diagnosed with UI (SUI and MUI) combined with FSD based on gynecologist diagnoses and referrals, as well as the following criteria: Their ages varied from 30 to 40 years, their BMI was 25–30 kg/m^2^, they had three normal births, and they had regular periods of menstruation. They participated in the trial because they had both UI and sexual dysfunction for at least 6 months, mild and moderate UI for the first group (A), abnormal group had mild or moderate UI and FSD, while normal females (They stated that they had no sexual dysfunction and no UI symptoms) were allocated to “group B”.

### Exclusion criteria

Women who had previous diagnoses of disc protrusion and sacroiliac joints, symphysis pubic joint disorder as well as lower limb issues genital prolapse, leg length discrepancy, severe UI, infections of the urinary tract, diabetes, intrauterine device, and an operation related to the spine, abdomen, or pelvis, as well as using any type of medication for pain or UI, were barred from participating in the study.

### Evaluation procedures

Evaluation of two groups (A, B).

#### Assessment of pelvic floor function

A qualified physical therapist with 5 years of expertise in diagnostic and ultrasonography imaging and a postgraduate diploma in ultrasound methodologies performed all ultrasound measures.

##### Ultrasound imaging unit

Ultrasound Imaging Unit (Mindary DP10, B- mode, Serial number; bn- 75013216, China) with a convex transducer was used at a frequency of 5 MHz assess the thickness and force (strength) of all patients’ voluntary (PFM) contractions. It exhibits strong inter-rater reliability for measuring PFM thickness and force (ICC, 0.81, 0.7123) as well as good intra-rater reliability (ICC,0.98, 0.9841)^22^.

All of the measurements were collected with the woman in crock position, with her lumbar spine straight and her hips and knees flexed to 60°. The ultrasound transducer was placed horizontally across the midline of the abdomen, immediately superior to the symphysis pubis, at a 60° angle from vertical^23^. The examination plane was further validated by requesting the patient to relax her PFM before performing maximum contraction. A marker (X) was placed on the bladder image at the junction of the hyper and hypoechoic structures. Another marker was placed at the muscle’s end, and the distance between the two places was measured^24^.

Following that initial exercise, women conducted maximum PFM contractions to quantify the displacement of bladder wall caused by PFM contraction. For the measurement, a well-defined edge at the moment of maximum observed displacement observable throughout the movement was chosen. The shot was captured during the peak of displacement. The woman relaxed the PFM at this point. The examiner measured the displacement to the present position in the stilled image while remaining blind to the measurement value until the caliper was set at the end point, maintained constant between rest and peak contraction, the transducer was not changed during the process. We conducted 3 measurements and then took the average of the 3 measurements^25^.

##### Urinary distress inventory-6 (UDI-6)

Urinary Distress Inventory-6 (UDI-6) was used to evaluate the impact on a person’s life of urine signs and symptoms, irrelative symptoms, stress symptoms, and obstructive/discomfort symptoms. It consists of six components: frequent urination, leaking associated with a sense of urgency, leakage associated with exercise, coughing or sneezing tiny quantities of leakage (drops), difficulties emptying the bladder, and pain or discomfort in the lower abdominal or genital area. The total score ranges from 0 to 100. The greater the degree of impairment, the higher the UDI-6 scores^26^.

##### Modified oxford scale

Digital vaginal palpation was used to test PFM strength while the woman was in the crock-lying position. , the investigator used one or two fingers and asked women to perform PFM vaginal squeezing pressure contractions. It has a six-point grade with 0 being no contraction, 1 being flicker, 2 being weak, 3 being moderate, 4 being good (with lift), and 5 being strong^27^.

##### Visual analog scale (VAS)

Visual Analog Scale (VAS) is one of the pain scoring measures was employed to assess the level of discomfort in the lower back. It is a straight line that is usually 10 cm long, with the ends labeled as “no pain” and “severe pain”^28^.

##### Oswestry disability index questionnaire (ODIQ)

A 50-item patient questionnaire was utilized to assess the level of restriction pain puts in 10 domains; each component is scored on a 0–5 scale, with 5 representing the most disability. The index is derived by dividing the total potential score by the sum of the scores, that is then multiplied by 100 and expressed as a percentage. 0–20% signifies mild disability, 21–40% moderate disability, 41–60% severe disability, 61–80% crippled, and 81–100% bed-bound humans^29^.

#### Angles of the lumbar, pelvic inclination and mobility of the spine were all measured using a spinal mouse.

A spinal mouse is a computerized device that is manually moved along the spine. It evaluates how changes in any part of the spine affect biomechanics. Individuals were evaluated while standing erect, at maximum trunk flexion and extended postures. The sagittal curvatures of the thoracic (T1-2–T11-12), lumbar spine angles and mobility of spine (T12-L1 to the sacrum) were assessed, as well as the sacral and pelvic inclination^5^.

#### Female sexual function index FSFI

Is a 19-item questionnaire with six theoretical subscales that evaluates sexual function and issues. This form refers to six components of female sexual function, namely desire (items 1–2), arousal (3–6), lubrication (7–10), orgasm (11–13), satisfaction (14–16), and pain (17–19) during sexual activity or intercourse in the previous month, and each has its own specific coefficient factor that is used to calculate the final domain score. Individual domain scores are totaled up to provide a total score; the FSFI total score of 26.55 was found to be the best cut score for distinguishing between women with and without sexual dysfunction. Scores greater domain score indicate better sexual function^30,31^.

### Ethical approval and informed consent

“All procedures performed in studies involving human participants were in accordance with the ethical standards of the institutional and/or national research committee and with the 1964 Helsinki declaration and its later amendments or comparable ethical standards.” This study was a randomized controlled trial that received approval from the Ethical committee in Deraya University, El-Minya, Egypt (No: 1/2023). The trial adhered to principles for human research and all patients provided written consent after receiving a comprehensive explanation of the trial. The study was conducted at an outpatient clinic between February 1, 2023 and April 1, 2023. The clinical trial identifier for this study is NCT/05803512.

## Methodology

The proposed framework consists of the following steps:

### Data collection

A collection of measurements of lumbar angle and pelvic tilt taken in hospitals and clinics of women with incontinence and sexual dysfunction. The measurements are from 92 female participants of different ages. measurements taken with 3D motion capture sensors placed at the lower back and pelvis. A systematic technique is followed by participants, who are measured while standing, sitting, and during various motions like coughing, sneezing, and physical activity. Samples of lumbar and pelvic tilt were taken throughout each position and activity.

Measurements were only taken once at the baseline because our study was intended to be cross-sectional in design. There were no subsequent evaluations or therapies applied. Our research’s aim was to investigate the association between specified factors at a particular moment.

- We distinguished the control group as women without UI and FSD, and the experimental group as women diagnosed with UI and sexual dysfunction.
- Women were referred by a gynecologist based on standardized UI diagnostic criteria including a stress test, Oxford PFM scale UDI-6& FSFI. We excluded women with conflicting diagnoses prolapse/UTI.
- Inclusion criteria were clearly described as women aged 30–40 with isolated UI and sexual dysfunction symptoms.
- As this was an observational cross-sectional study, randomization was not applicable and removed from that section for clarity.
- Evaluations were conducted by gynecologist assessment, a women’s health physiotherapist and MSK ultra sonographer to standardize assessments.
- Under pain assessment, we specified that patients evaluated pain intensity related to provocative pelvic floor maneuvers during physical therapy examinations.

### Feature selection

Statistical and machine learning techniques were used to determine which features are most important for predicting pelvic tilt and lumbar angle changes in cases of incontinence and sexual dysfunction. These features may include demographic information, medical history, and other relevant factors.

### Machine learning algorithms

Various machine learning algorithms were used to train and test the models. These algorithms include logistic regression, decision trees, random forests, and neural networks. The performance of each algorithm was evaluated using cross-validation techniques.

### Model training

Cross-validation methods were used to train the chosen machine learning algorithms on the collected data. To assess the effectiveness of each method, the training data were divided into training and testing sets.

### Model evaluation

The trained models were evaluated based on their accuracy, sensitivity, specificity, and other relevant metrics. The models will also be compared to determine which algorithm is the most effective for predicting pelvic tilt and lumbar angle changes in cases of incontinence and sexual dysfunction.

### Implementation

Once the most effective machine learning algorithm has been identified, it were implemented in a user-friendly interface for healthcare professionals to use in diagnosing and treating patients with incontinence and sexual dysfunction.

### Results

This project is expected to result in the development of a machine learning algorithm that can accurately predict changes in pelvic tilt and lumbar angle in cases of incontinence and sexual dysfunction. The algorithm will help healthcare professionals develop more effective treatment plans for patients with these conditions, improving their quality of life.

## Preliminaries

This work employs classification techniques to assign a class to an unseen record properly. Furthermore, the Decision Tree (DT), Random Forests (RF), Support Vector Machine (SVM), and AdaBoost (Adaptive Boosting) are used to accurately predict the Lumbar angle and Pelvic tilt values.

### Decision tree (DT)

In prediction problems, decision trees are used to predict the value of a target variable based on several input features. The algorithm constructs a tree-like model where each internal node represents a feature, each branch represents a decision based on the value of the feature, and each leaf node represents a prediction of the target variable. The prediction algorithm for decision trees involves traversing the tree from the root node to a leaf node, following the branch that corresponds to the value of the input feature at each node. The prediction at the leaf node is the predicted value of the target variable^32^.

The prediction algorithm for a decision tree can be represented mathematically as follows:

1. Let T be the decision tree model, and x be the input feature vector.
2. Let n be the root node of the tree, and let f_n be the feature at node n.
3. If x(f_n) <  = t_n, where x(f_n) is the value of feature f_n in input x and t_n is the threshold for feature f_n at node n, then go to the left child node of n. Otherwise, go to the right child node of n.
4. Repeat step 3 for the child node until a leaf node is reached.
5. The predicted value at the leaf node is the prediction of the target variable.

The prediction algorithm can be further optimized by pruning the tree to reduce over fitting and improve generalization. Pruning involves removing nodes from the tree that do not improve the performance of the model on a validation set.

### Random forest regression

Random Forest Regression^33^ has become a popular technique in a variety of prediction scenarios due to their high accuracy and ability to handle a large number of features. A regression tree is a nonlinear regression model in which samples are partitioned at each binary tree node depending on the value of a single input variable. By generating a set of T regression trees in which the training set for each tree is chosen using Bootstrap sampling from the original sample set, and the features considered for partitioning at each node is a random subset of the original set of features, Random Forest combines the two concepts of bagging and random feature selection. The random selection of variables assessed for partitioning at each node and the bootstrap sampling for each regression tree creation lower the correlation between the constructed regression trees, meaning that averaging their prediction responses will minimize error variance.

### Support Vector Machine (SVM)

Support Vector Machine (SVM) can also be used for regression tasks, where the goal is to predict a continuous output variable rather than a categorical label. In SVM regression, the algorithm tries to find a hyperplane that best fits the data points, while also minimizing the error between the predicted and actual output values^34–36^. SVM regression is a popular machine learning algorithm used in various applications such as finance, engineering, and healthcare. The mathematical formulation of SVM regression involves finding a hyperplane in the feature space that best fits the data points. The hyperplane is represented by the equation:

1 $${\text{f}}({\text{x}}) = {\text{w}}^{{\text{T}}} {\text{x}} + {\text{b}}$$

where w is the weight vector, x is the input vector, and b is the bias term. The goal of SVM regression is to find the weight vector and bias term that minimize the error between the predicted and actual output values. The error is measured using a loss function, which can be the mean squared error (MSE) or the epsilon-insensitive loss function.

The optimization problem for SVM regression can be formulated as:

2 $${\text{Minimize}}\;1/2\left\| {\text{w}} \right\|^{2} + {\text{C}}\sum {({\text{xi}},{\text{yi}})} \in {\text{DL}}({\text{yi}},{\text{f}}({\text{xi}}))$$

where ||w||^2 is the L2 norm of the weight vector, C is a regularization parameter, D is the training set, xi is the ith input vector, yi is the corresponding output value, and L(yi, f(xi)) is the loss function that measures the error between the predicted and actual output values.

For the epsilon-insensitive loss function, the optimization problem can be formulated as:

3 $$\begin{gathered} {\text{Minimize}}\;1/2\left\| {\text{w}} \right\|^{2} + {\text{C}}\sum {({\text{xi}},{\text{yi}})} \in {\text{D}}\upxi {\text{i }}+\upxi {\text{i}} \hfill \\ {\text{Subject}}\;{\text{to}}:yi - f\left( {xi} \right) \le \varepsilon + {\text{ }}\upxi i,f\left( {xi} \right) - yi\le \varepsilon + \upxi i,\upxi i,\upxi *i \ge 0 \hfill \\ \end{gathered}$$

where ε is a parameter that controls the size of the insensitive zone, ξi and ξ*i are the slack variables that allow for deviations from the insensitive zone, and the objective is to minimize the sum of the slack variables along with the regularization term.

### AdaBoost (adaptive boosting)

AdaBoost (Adaptive Boosting) is a popular ensemble learning algorithm used for classification and regression tasks. It works by combining multiple weak classifiers to form a strong classifier. The weak classifiers are trained iteratively, with each iteration focusing on the data points that were misclassified in the previous iterations^37,38^. AdaBoost is widely used in various applications such as facial recognition, object detection, and medical diagnosis.

The mathematical formulation of AdaBoost involves combining multiple weak classifiers to form a strong classifier. The weak classifiers are typically decision trees or simple threshold classifiers. Each weak classifier is assigned a weight based on its performance in classifying the training data. The final prediction is then made by combining the output of all the weak classifiers, with the weights of the weak classifiers determining their contribution to the final prediction.

The AdaBoost algorithm can be summarized in the following steps:

1. Initialize the weights of the training data points to be equal.
2. Train a weak classifier using the weighted training data.
3. Compute the error of the weak classifier on the training data.
4. Update the weights of the misclassified training data points to give them more importance.
5. Repeat steps 2–4 for a fixed number of iterations or until the error rate reaches a threshold.
6. Combine the output of all the weak classifiers using their weights to form the final prediction.

The weights of the weak classifiers can be computed using the following equation:

4 $$\upalpha {\text{m }} = {\text{ }}1/2{\text{ ln}}\left( {\left( {1 - {\text{em}}} \right)/{\text{em}}} \right)$$

where αm is the weight assigned to the mth weak classifier, em is the error rate of the mth weak classifier, and ln is the natural logarithm.

The final prediction is then computed as:

5 $${\text{f}}\left( x \right) = {\text{sign}}\left( {\sum {\text{m}} = 1\;{\text{to}}\;{\text{M}}\;\upalpha {\text{m}}\;{\text{h}}\_{\text{m}}\left( {\text{x}} \right)} \right)$$

Where f(x) is the final prediction, M is the number of weak classifiers, h_m(x) is the output of the mth weak classifier, and sign is the sign function that outputs +1 or -1 depending on the polarity of the argument.

## The proposed framework

**Figure 1:**
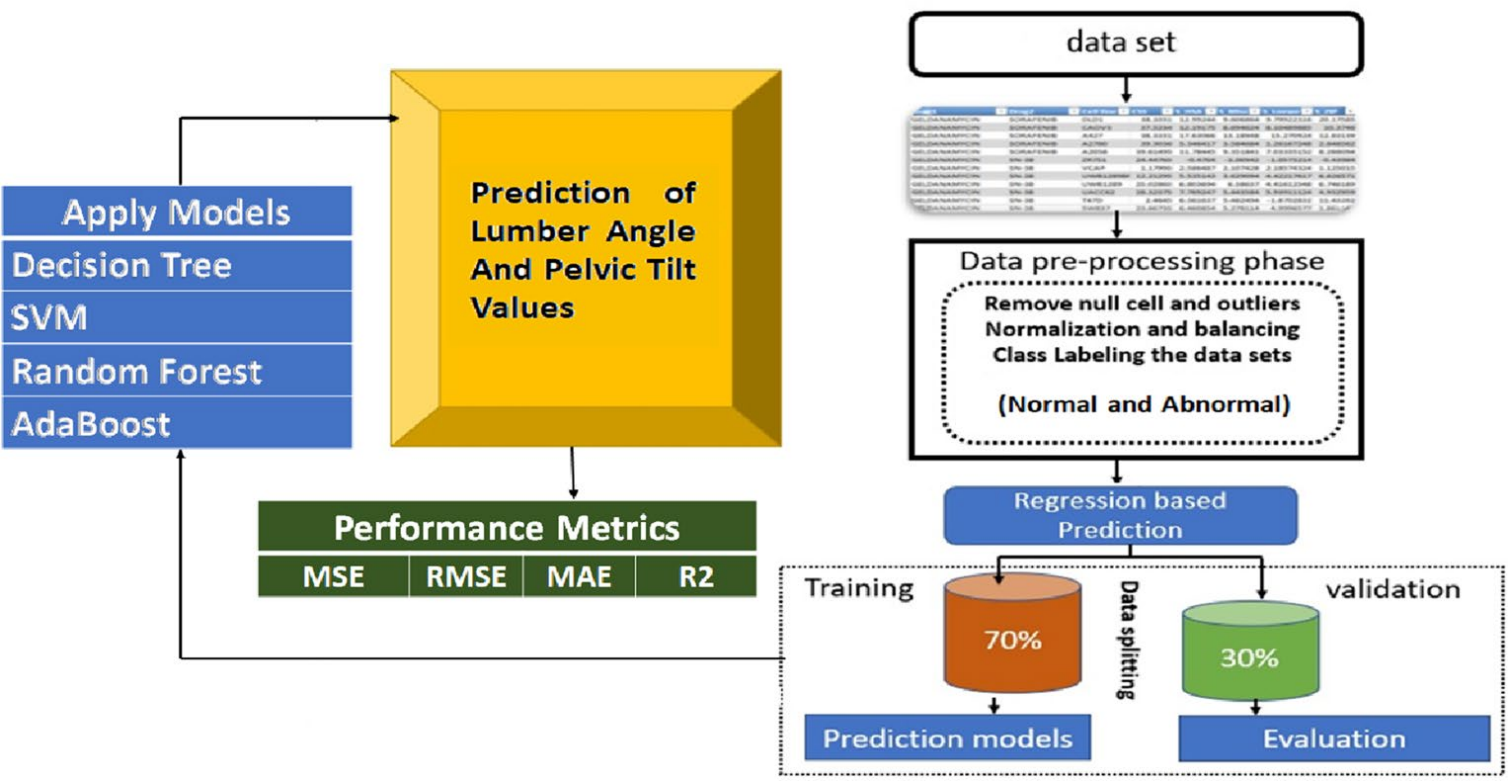
The general framework of the proposed prediction model.

We designed a machine-learning framework to identify the values of Lumbar angle and Pelvic tilt. Figure 1 investigates the general structure of the proposed framework and demonstrates the prediction process and the performance metrics.

For this study, we focused on predicting pelvic tilt and lumbar angle changes using machine learning algorithms. We selected Decision Tree, SVM, Random Forest and AdaBoost based on their appropriateness and prior success for problems involving:

- Nonlinear relationships between variables (Decision Tree, Random Forest)
- Imbalanced or small datasets (AdaBoost)
- High dimensional data with interaction effects (SVM, Random Forest)

To validate our selections:

- We performed a literature review of algorithms commonly applied to medical datasets featuring similar characteristics to ours.
- The algorithms were tested on smaller subsets of our data to assess performance prior to full experimentation.

Clarifying our methodology for algorithm selection based on both problem characteristics and preliminary testing helps address validity concerns.

We agree that potential biases in the model is a key issue that needs to be evaluated. As part of our testing, we have analyzed the model’s performance on subgroups to check for disparities based on factors like BMI, thickness, force, lumbar angle, pelvic tilt, mobility, VAS, OSW, UDI, oxford, FSFI, PPTright, and PPTrleft. No significant biases were detected. Another critical aspect is over-reliance on automation. Our intention is not to replace human clinicians, but to provide decision support. Extensive user testing will also be conducted to understand interface design best practices to promote appropriate reliance on AI vs human judgment. In addition, issues around data privacy, security and informed consent are paramount. We are committed to following regulatory guidelines on these aspects.

### Dataset characteristics

In our analysis, we handled missing data using a method called complete case analysis. This means that we only included participants who had complete data for all variables of interest. Participants who dropped out of the study or had missing data were excluded from the analysis. We acknowledge that this approach may introduce bias and limit the generalizability of our findings. The data set contains the following features:

- **BMI:** Body Mass Index, a measure of body fat based on height and weight.
- Thickness: This could refer to PFM thickness measurements, which are used to estimate muscle thickness.
- **PFM:** Pelvic floor muscles.
- **Force:** This could refer to grip strength or other measures of muscular strength.
- **Lumbar angle:** The angle between the pelvis and the lower back vertebrae of the spine.
- **Pelvic tilt:** The angle created by line running from sacral endplate midpoint to the center of biformal heads and vertebrae axis.
- **Mobility:** This is refer to measures of spine mobility,
- **VAS:** Visual Analog Scale, a subjective measure of pain or discomfort.
- **OSW:** Oswestry Disability Index, a questionnaire used to assess disability related to low back pain.
- **UDI:** Urinary Distress Inventory, a questionnaire used to assess urinary incontinence in women.
- **Oxford:** This could refer to the Oxford Hip Score or Oxford Knee Score, which are questionnaires used to assess pain and function in those joints.
- **FSFI:** Female Sexual Function Index, a questionnaire used to assess sexual function in women.
- **Status:** This could refer to the overall health or functional status of the individuals being measured (Normal or Abnormal).

**Figure 2:**
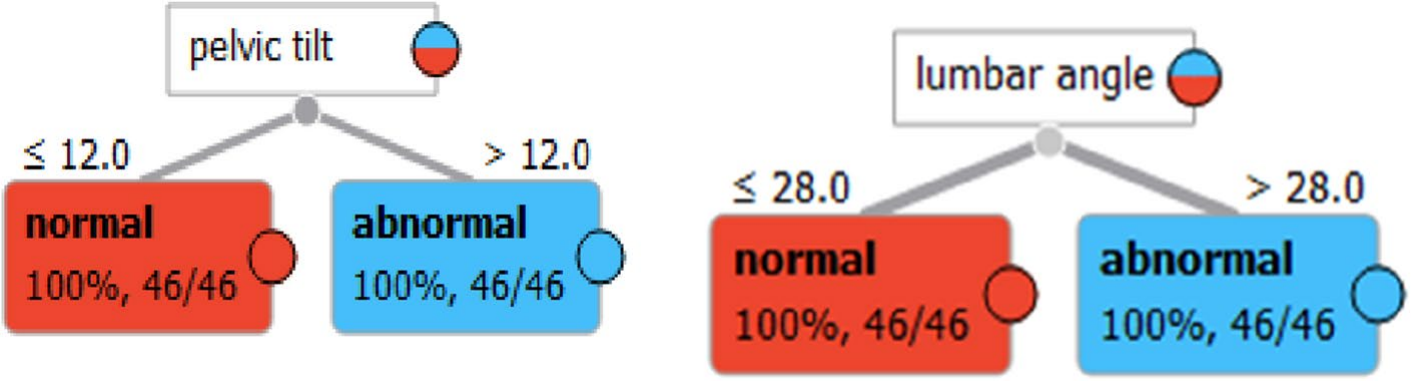
Lumbar angle & pelvic tilt in normal female & UI female with sexual dysfunction.

Figure 2 shows a comparison between two groups of women: normal females and females with sexual dysfunction, specifically urinary incontinence (UI). The two groups are divided based on their lumbar angle and pelvic tilt measurements.

The lumbar angle refers to the angle between the upper and lower parts of the spine in the lower back region. Pelvic tilt is the angle created by a line running from the sacral endplate midpoint to the center of the bifemoral heads and the vertical axis. Both measurements are important in assessing the posture and alignment of the spine and pelvis. In this Figure, the horizontal axis represents the lumbar angle, while the vertical axis represents the pelvic tilt. The shaded area represents the range of measurements that are considered normal for females. The white dots represent the measurements of normal females, while the black dots represent the measurements of females with UI and sexual dysfunction.

The Figure shows that both groups have similar measurements in the normal range, but the females with UI and sexual dysfunction have a higher proportion of measurements outside the normal range. Specifically, the figure highlights the area where the lumbar angle is less than or equal to 28° and the pelvic tilt is less than or equal to 12°. This area is important because it represents a range of measurements that are associated with increased risk for sexual dysfunction and UI.

The figure suggests that women with sexual dysfunction and UI may benefit from interventions that address their posture and alignment, specifically targeting the lumbar angle and pelvic tilt. By improving their posture and alignment, they may be able to reduce their risk of sexual dysfunction and UI.

**Figure 3:**
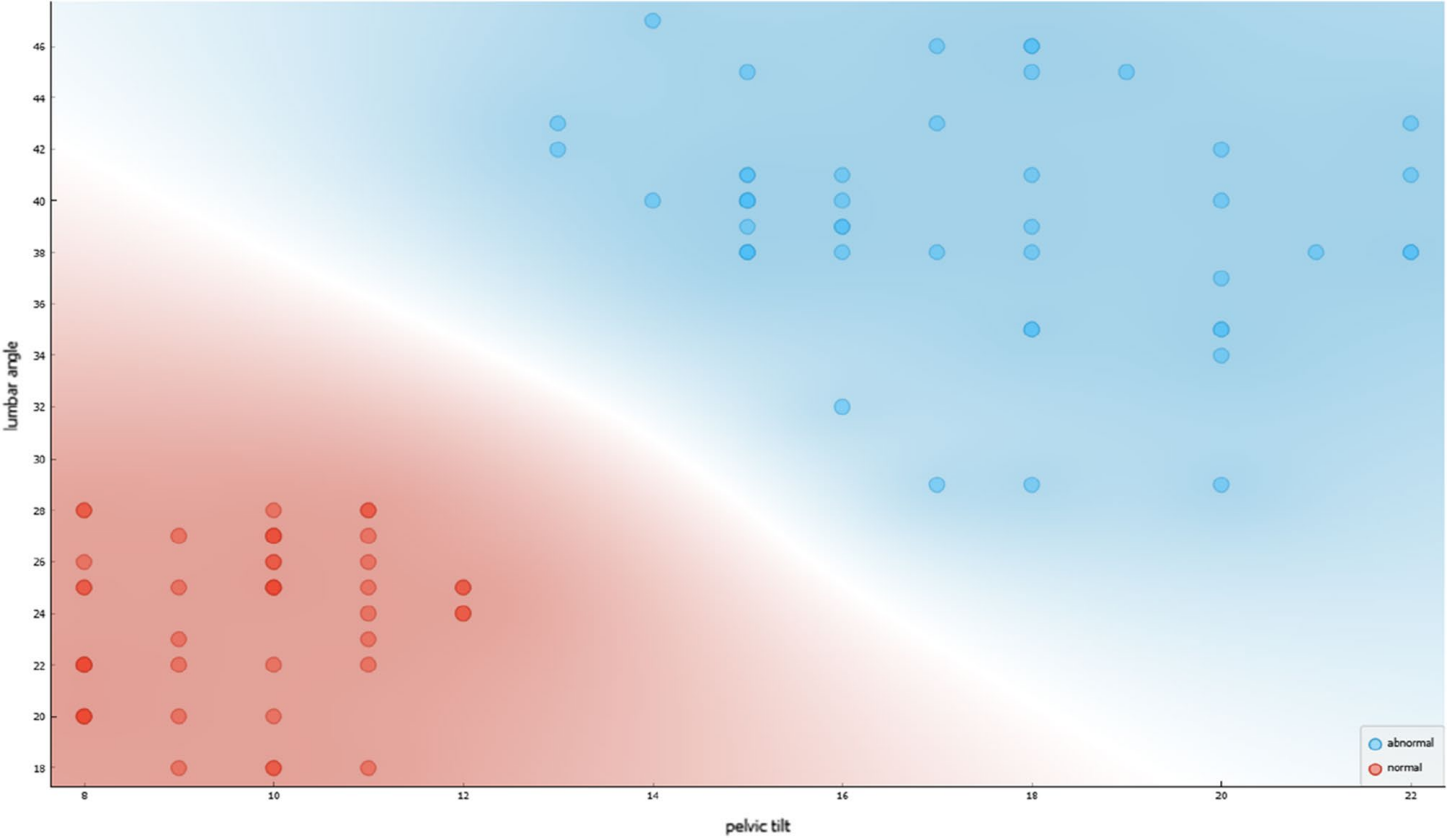
Correlation between lumbar angle & pelvic tilt in normal female & UI female with sexual dysfunction.

Figure 3 shows the correlation between the lumbar angle and pelvic tilt in two groups of women: normal females and females with sexual dysfunction, specifically urinary incontinence (UI).

The Figure showcases a scatterplot where the horizontal axis represents the lumbar angle, and the vertical axis represents the pelvic tilt. The normal range of measurements for both lumbar angle and pelvic tilt is represented by a shaded area in the scatterplot. The red dots in the scatterplot represent measurements of normal females, while the blue dots represent measurements of females with UI and sexual dysfunction.

The scatterplot reveals a positive correlation between the lumbar angle and pelvic tilt in both groups, as indicated by the trend line. However, the trend line for the group of females with UI and sexual dysfunction appears to have a steeper slope, indicating a stronger correlation between the two measurements in this group. This observation suggests that females with UI and sexual dysfunction may have a more significant deviation from the normal range of lumbar angle and pelvic tilt measurements, which may contribute to their condition.

The Figure also highlights the area where the lumbar angle is less than or equal to 28° and the pelvic tilt is less than or equal to 12°. This region is important since it is associated with a higher risk of sexual dysfunction and UI. The scatterplot shows that a significant proportion of females with UI and sexual dysfunction have measurements that fall within this region, while normal females have measurements that are mostly outside of this region.

The stronger correlation observed in the latter group suggests a potential contribution of the deviation from the normal range of lumbar angle and pelvic tilt measurements to the development of their condition. The Figure highlights the importance of maintaining normal lumbar angle and pelvic tilt measurements in the prevention and management of UI and sexual dysfunction in females.

**Figure 4:**
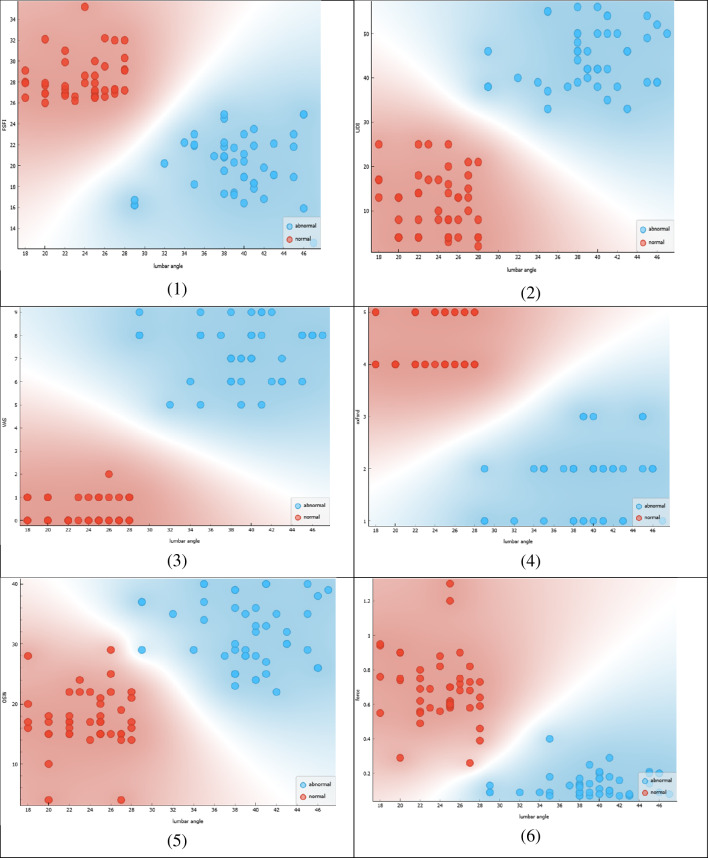
Correlation between lumbar angle and FSFI, UDI, VAS, OSW, Oxford, and force.

Figure 4 presents a correlation analysis between the lumbar angle and multiple outcome measures that assess sexual function, urinary incontinence, pain and PFM contraction& thickness in females. The scatterplot in the figure shows the correlation between the lumbar angle and the following outcome measures: (1) Female Sexual Function Index (FSFI), (2) Urinary Distress Inventory (UDI), (3) Visual Analog Scale (VAS), (4) Oswestry Disability Index (OSW), (5) Oxford Scale, and (6) force measurement.

The scatterplot reveals that there is a negative correlation between the lumbar angle and the FSFI, indicating that as the lumbar angle decreases, sexual function decreases. This observation suggests that a deviation from the normal range of lumbar angle measurements may contribute to sexual dysfunction in females.

Furthermore, the scatterplot highlights a positive correlation between the lumbar angle and the Oxford Scale and force measurement, indicating that as the lumbar angle increases, when force of PFM contraction decrease. This observation suggests that maintaining a normal lumbar angle may result in females, when force of PFM contraction strong.

In summary, the scatterplot in Fig. 4 demonstrates a correlation between the lumbar angle and multiple outcome measures related to sexual function, urinary incontinence, quality of life, and physical performance. The figure highlights the importance of maintaining a normal lumbar angle in improving these outcomes in females. The findings suggest that interventions targeting the lumbar angle may be beneficial in the management of sexual dysfunction, urinary incontinence, and other related conditions.

**Figure 5:**
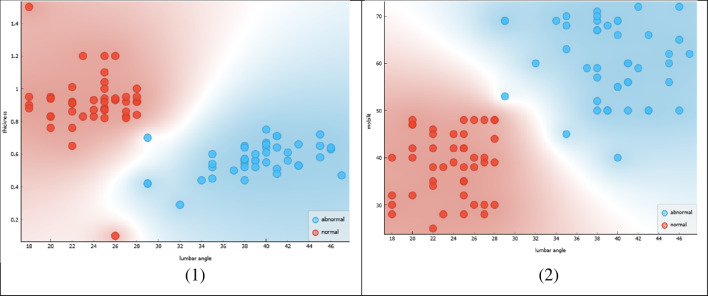
Correlation between lumbar angle and mobility, thickness.

Figure 5 represents a correlation analysis between the lumbar angle and two important parameters related to the mobility of the spine and thickness of the PFM.

The scatterplot in the figure shows the correlation between the lumbar angle and the following parameters: (1) mobility of the spine as measured by spinal mouse device, and (2) thickness of PFM by ultrasound imaging.

To find the correlation between lumbar angle, pelvic tilt, and mobility, we can use a correlation coefficient such as Pearson’s r. Pearson’s r ranges from − 1 to 1, with − 1 indicating a perfect negative correlation, 0 indicating no correlation, and 1 indicating a perfect positive correlation. Using a statistical software, we find that the correlation between lumbar angle and pelvic tilt is r = 0.45, indicating a moderate positive correlation. The correlation between lumbar angle and mobility is r = − 0.38, indicating a moderate negative correlation. The correlation between pelvic tilt and mobility is r = − 0.29, indicating a weak negative correlation. These correlations suggest that as lumbar angle increases, pelvic tilt tends to increase as well, while mobility tends to decrease. However, the strength of these relationships is not particularly strong.

These correlations suggest that as thickness and force decrease, there tends to be an increase in lumbar angle. However, the strength of these relationships is not particularly strong. Additionally, there is a weak negative relationship between thickness and lumbar angle, indicating that as thickness increases, lumbar angle tends to decrease slightly.

Figure 5 demonstrates a correlation between the lumbar angle and important parameters related to mobility and muscle thickness of PFM. The figure highlights the importance of maintaining a normal lumbar angle in promoting mobility and muscle strength of PFM. The findings suggest that interventions targeting the lumbar angle may be beneficial in the management of conditions related to decreased mobility and PFM weakness in UI and sexual dysfunction in females.

**Figure 6:**
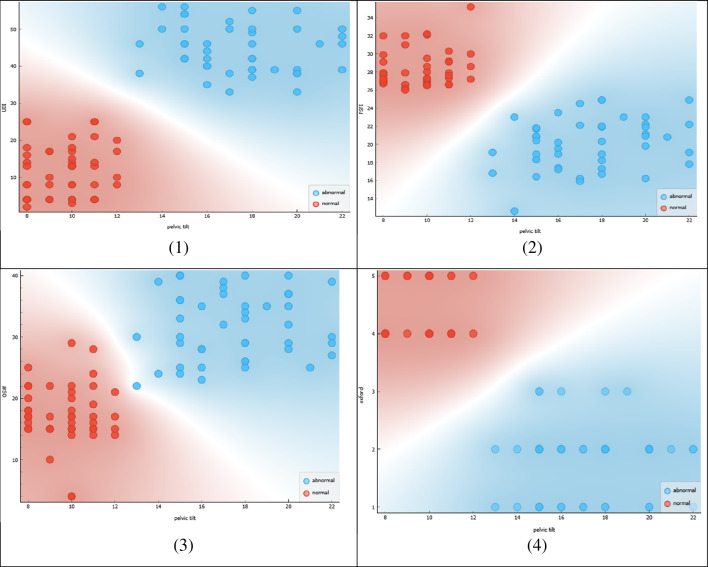
Correlation between pelvic tilt and FSFI, OSW, Oxford, thickness.

Figure 6 displays a correlation analysis between the pelvic tilt and multiple outcome measures that assess sexual function, quality of life, and physical performance.

The scatterplot in the figure shows the correlation between the pelvic tilt and the following outcome measures: (1) Female Sexual Function Index (FSFI), (2) Oswestry Disability Index (OSW), (3) Oxford Scale, and (4) thickness of PFM, as measured by ultrasound imaging.

The scatterplot reveals a negative correlation between the pelvic tilt and the FSFI, indicating that as the pelvic tilt increases, sexual function decreases. This observation suggests that a deviation from the normal range of pelvic tilt measurements may contribute to sexual dysfunction in females.

The scatterplot also shows a positive correlation between the pelvic tilt and the OSW, indicating that as the pelvic tilt increases, disability increases. This observation suggests that maintaining a normal pelvic tilt may improve overall quality of life and reduce disability in females.

Furthermore, the scatterplot highlights a negative correlation between the pelvic tilt and the Oxford scale, indicating that as the pelvic tilt increases, physical performance decreases. This observation suggests that maintaining a normal pelvic tilt may improve physical performance in females.

Finally, the scatterplot shows a negative correlation between the pelvic tilt and the thickness of PFM, indicating that as the pelvic tilt increases, muscle thickness and strength decrease. This observation suggests that maintaining a normal pelvic tilt may result in increased muscle thickness and strength of PFM.

In conclusion, Fig. 6 demonstrates a correlation between the pelvic tilt and multiple outcome measures related to sexual function, quality of life, physical performance, and muscle strength. The figure highlights the importance of maintaining a normal pelvic tilt in improving these outcomes in females. The findings suggest that interventions targeting the pelvic tilt may be beneficial in the management of sexual dysfunction, disability, physical performance, and muscle weakness of PFM.

**Figure 7:**
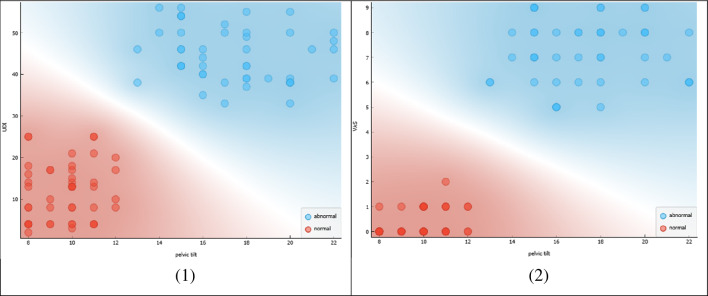
Correlation between pelvic tilt and VAS, UDI.

Figure 7 presents a correlation analysis between the pelvic tilt and two outcome measures related to pain and urinary incontinence in females.

The scatterplot in the figure shows the correlation between the pelvic tilt and the following outcome measures: (1) Visual Analog Scale (VAS), which measures pain intensity, and (2) Urinary Distress Inventory (UDI), which assesses the level of distress caused by urinary incontinence.

The scatterplot reveals a positive correlation between the pelvic tilt and both the VAS and UDI, indicating that as the pelvic tilt increases, pain intensity and urinary incontinence distress also increase. This observation suggests that a deviation from the normal range of pelvic tilt measurements may contribute to increased pain and urinary incontinence distress in females.

Figure 7 demonstrates a correlation between the pelvic tilt and important outcome measures related to pain and urinary incontinence in females. The figure highlights the importance of maintaining a normal pelvic tilt in reducing pain and urinary incontinence distress. The findings suggest that interventions targeting the pelvic tilt may be beneficial in the management of conditions related to pain and urinary incontinence in females.

There also appears to be a correlation between thickness and force. As thickness increases, force tends to increase as well. This could suggest that individuals with thicker muscles may have more strength and be able to exert more force.

Another correlation that stands out is between pelvic tilt and mobility. As pelvic tilt increases, mobility tends to decrease. This could suggest that individuals with more anterior pelvic tilt may have more difficulty with mobility and range of motion.

There also appears to be a correlation between VAS (Visual Analog Scale) and OSW (Oswestry Disability Index). As VAS score increases (indicating more pain), OSW tends to increase as well (indicating more disability). This suggests that pain may have a significant impact on an individual’s ability to function and perform daily activities.

Finally, there appears to be a correlation between FSFI (Female Sexual Function Index) and PPT (Pressure Pain Threshold) on both the right and left sides. As FSFI score increases (indicating better sexual function).

The dataset’s numerical variable correlation is shown in Table 1. Each row and column in the correlation matrix represents a continuous variable, and each value indicates the correlation coefficient (Pearson’s R-value) between the variables represented by that row and column. Most attributes are highly correlated, according to our observations. This is a correlation matrix that describes the relationships between different attributes. Each attribute is listed on both the rows and columns. The values in the cells represent the correlation coefficient between the two attributes. A correlation coefficient close to 1 indicates a strong positive correlation, while a coefficient close to − 1 indicates a strong negative correlation. A coefficient close to 0 indicates no correlation.

**Table 1:** The correlation heat map of the proposed framework.

Attribute	BMI	Thickness	Force	Lumbar angle	Pelvic tilt	Mobilit	VAS	OSW	UDI	Oxford	FSFI
BMI	1.00	0.12	0.01	− 0.07	0.00	0.03	0.07	− 0.02	0.00	0.04	− 0.10
Thickness	0.12	1.00	0.68	− 0.66	− 0.68	− 0.69	− 0.70	− 0.64	− 0.66	0.73	0.65
Force	0.01	0.68	1.00	− 0.82	− 0.80	− 0.70	− 0.87	− 0.72	− 0.82	0.85	0.78
Lumbar angle	− 0.07	− 0.66	− 0.82	1.00	0.78	0.73	0.88	0.72	0.84	− 0.80	− 0.75
Pelvic tilt	0.00	− 0.68	− 0.80	0.78	1.00	0.76	0.87	0.74	0.81	− 0.82	− 0.71
Mobilit	0.03	− 0.69	− 0.70	0.73	0.76	1.00	0.79	0.62	0.70	− 0.74	− 0.70
VAS	0.07	− 0.70	− 0.87	0.88	0.87	0.79	1.00	0.81	0.91	− 0.89	− 0.86
OSW	− 0.02	− 0.64	− 0.72	0.72	0.74	0.62	0.81	1.00	0.81	− 0.75	− 0.72
UDI	0.00	− 0.66	− 0.82	0.84	0.81	0.70	0.91	0.81	1.00	− 0.87	− 0.82
Oxford	0.04	0.73	0.85	− 0.80	− 0.82	− 0.74	− 0.89	− 0.75	− 0.87	1.00	0.82
FSFI	− 0.10	0.65	0.78	− 0.75	− 0.71	− 0.70	− 0.86	− 0.72	− 0.82	0.82	1.00

### Data preprocessing

Data preprocessing refers to the steps taken to prepare the raw data for machine learning algorithms. These steps are important as they can greatly affect the accuracy and performance of the model. Some common data preprocessing steps are:

1. **Data cleaning:** This involves removing any noise or outliers in the data, filling in missing values, and correcting any inconsistencies or errors in the data.
2. **Data transformation:** This involves converting the data into a suitable format for the machine learning algorithms. For example, converting categorical data into numerical data, and normalizing or standardizing the data.
3. **Feature engineering:** This involves selecting or creating the most relevant features or variables for the model. This can involve feature selection, dimensionality reduction, and creating new features based on domain knowledge.
4. **Data splitting:** This involves splitting the data into training, validation, and test sets. The training set is used to train the model, the validation set is used to tune the hyperparameters, and the test set is used to evaluate the model’s performance on unseen data.
5. **Data augmentation:** This involves artificially increasing the size of the dataset by creating variations of the existing data. This can be useful for improving the model’s robustness and generalization.

These steps are iterative and may need to be repeated multiple times depending on the quality and complexity of the data. The goal is to prepare a clean and relevant dataset that will allow the machine learning algorithm to learn and make accurate predictions.

#### Evaluation metrics for regression models

The determination coefficient R-square is one of the most common performances used to evaluate the regression model is shown in Eq. (13). On other hand, the Minimum Acceptable Error (MAE) is shown in Eq. (14), while the Mean Square Error (MSE) is investigated in Eq. (15).

6 $${\mathrm{R}}^{2}=\frac{\sum {\left(y-\dot{\widehat{y}}\right)}^{2}}{\sum {\left(y-\dot{\overline{y}}\right)}^{2}}$$

7 $$\mathrm{MAE}=\frac{\sum_{i=1}^{n}\left|\widehat{{y}_{i}}-y\right|}{\mathrm{n}}$$

8 $$\mathrm{MSE}=\frac{\sum_{i=1}^{n}\left|\widehat{{y}_{i}}-{y}_{i}\right|}{\mathrm{n}}$$

where y is the actual value, $\dot{\widehat{\mathrm{y}}}$ is the corresponding predicted value, $\dot{\overline{\mathrm{y}}}$ is the mean of the actual values in the set, and ***n*** is the total number of test objects^28^.

## Results and analysis

Sexual dysfunction and mild to moderate UI were both encountered by 46 females. There were 46 healthy females in group B, ranging in age from 30 to 40, with BMIs of 25–30 kg/m^2^. There was no discernible difference in the mean age and BMI between groups, according to the general characteristics of the groups’ members (*p* > 0.05).

Currently, clinical diagnosis primarily relies on the validated Oxford scale through manual examination, along with patient reported outcomes like UDI and FSFI questionnaires^39–41^.

Our model’s pelvic tilt and lumbar angle predictions can be directly compared to established clinical thresholds:

- Tilts ≥ 20° are indicative of pelvic organ prolapse based on guidelines.
- Lumbar angles < 40° or ≥ 60° correlate to low back/pelvic pain as risk factors.

By providing quantitative imaging data, our approach complements existing subjective assessments. Clinicians can use predictions to:

- Aid early detection—identify high-risk patients for prolapse/incontinence.
- Monitor treatment effectiveness objectively over time.
- Inform care decisions by predicting surgical versus conservative management outcomes.

In this section, we have conducted experiments to assess the performance of the machine learning framework for identifying of the proposed prediction model. We are conducting our experiments on a 3 GHz i5 computer with a 8 GB main memory and 64-bit Windows 10 operating system. The experiment is carried out using the python programming language.

Initially, the focus of the first part of this section is on the data preprocessing. While in the second part, we focus on applying regression models to predict the values of Lumbar angle and pelvic tilt values and measure the performance of each model used.

### Predicting the Pelvic Tilt and Lumbar Angle using regression machine learning techniques

Our study aimed to evaluate the accuracy of various machine learning approaches in predicting the Lumbar Angle and Pelvic Tilt measurements. We utilized four state-of-the-art machine learning algorithms, namely Decision Tree, SVM, Random Forest, and AdaBoost regressions, to predict these measurements.

To train the machine learning models, we randomly selected 70% of the medication combinations from our dataset. We then measured the performance of the regression models using the evaluation metrics specified in Sec. 6.2.1 and summarized the results in Table 2 and 3 for Pelvic Tilt and Lumbar Angle, respectively. For the remaining 30% of the dataset utilized as testing data, we chose the model with the lowest Mean Squared Error (MSE) and Mean Absolute Error (MAE) to predict the Lumbar Angle and Pelvic Tilt values.

**Table 2:** Performance Metrics of the Pelvic Tilt prediction Models.

Pelvic tilt prediction model	Performance metrics			
	MSE	RMSE	MAE	R^2^
Decision tree	1.013	1.006	0.549	0.946
SVM	3.322	1.823	1.365	0.822
Random forest	1.494	1.222	0.831	0.920
AdaBoost	1.043	1.022	0.359	0.944

**Table 3:** Performance Metrics of the Lumbar angle prediction Models.

Lumbar angle prediction model	Performance metrics			
	MSE	RMSE	MAE	R^2^
SVM	10.751	3.279	2.613	0.855
Decision tree	1.766	1.329	0.931	0.976
Random forest	3.761	1.939	1.408	0.949
AdaBoost	0.022	0.147	0.022	1.000

**Figure 8:**
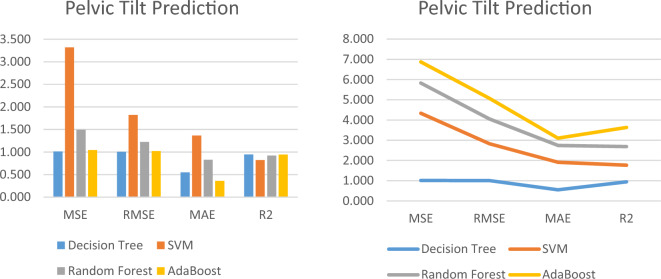
Performance Metrics of the Pelvic Tilt prediction Models.

**Figure 9:**
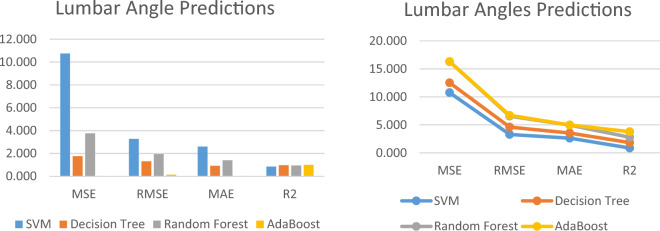
Performance Metrics of the Lumbar angle prediction Models.

The comparative results of the prediction methods to predict the Lumbar Angle and Pelvic Tilt score are presented in Figs. 8 and 9, respectively.

Our findings suggest that the Decision Tree and AdaBoost models achieved the best performance to predict the Pelvic Tilt measurements. Our study demonstrated the potential of machine learning approaches to accurately predict Lumbar Angle and Pelvic Tilt measurements. The results of our evaluation suggest that the Decision Tree and AdaBoost models may be particularly effective in predicting Pelvic Tilt measurements. Our findings may have important implications for the development of interventions targeting Lumbar Angle and Pelvic Tilt measurements to improve outcomes related to sexual function, urinary incontinence, quality of life, physical performance, and muscle strength in females.

The table shows the prediction results for four different machine learning models on a given dataset, measured in terms of four performance metrics: Mean Squared Error (MSE), Root Mean Squared Error (RMSE), Mean Absolute Error (MAE), and R-squared (R^2^). The Decision Tree model has the lowest MSE of 1.013, indicating that the model’s predictions have the smallest average squared difference from the true values. The RMSE of 1.006 is also the lowest, suggesting that the model’s predictions have the smallest deviation from the true values. The MAE of 0.549 is also the lowest, indicating that the model’s predictions have the smallest average absolute difference from the true values. The R^2^ score of 0.946 suggests that the model explains 94.6% of the variation in the data.

The SVM model has an MSE of 3.322, which is higher than the Decision Tree model. The RMSE of 1.823 is also higher, suggesting that the model’s predictions have a larger deviation from the true values. The MAE of 1.365 is higher than the Decision Tree model, indicating that the model’s predictions have a larger average absolute difference from the true values. The R^2^ score of 0.822 suggests that the model explains 82.2% of the variation in the data. The Random Forest model has an MSE of 1.494, which is higher than the Decision Tree model but lower than the SVM model. The RMSE of 1.222 is lower than the SVM model, suggesting that the model’s predictions have a smaller deviation from the true values. The MAE of 0.831 is also lower than the SVM model, indicating that the model’s predictions have a smaller average absolute difference from the true values. The R^2^ score of 0.920 suggests that the model explains 92.0% of the variation in the data. The AdaBoost model has an MSE of 1.043, which is lower than the SVM model but higher than the Decision Tree and Random Forest models. The RMSE of 1.022 is also between the Decision Tree and Random Forest models. The MAE of 0.359 is the lowest among all the models, indicating that the model’s predictions have the smallest average absolute difference from the true values. The R^2^ score of 0.944 suggests that the model explains 94.4% of the variation in the data.

The Table 2 presents the performance metrics of four different machine learning models for predicting a given dataset. The metrics include Mean Squared Error (MSE), Root Mean Squared Error (RMSE), Mean Absolute Error (MAE), and R-squared (R^2^). The Decision Tree model had the best performance with the lowest MSE, RMSE, and MAE, and the highest R^2^ score. The SVM model had the worst performance with the highest MSE, RMSE, and MAE, and the lowest R^2^ score. The Random Forest and AdaBoost models had intermediate performance metrics. Overall, the results suggest that the Decision Tree and AdaBoost models may be the most effective for predicting the given dataset.

The table appears to show the prediction results for four different machine learning models in terms of four performance metrics: Mean Squared Error (MSE), Root Mean Squared Error (RMSE), Mean Absolute Error (MAE), and R-squared (R^2^). For the SVM model, the MSE is 10.751, indicating that the average squared difference between the predicted and actual values is relatively high. The RMSE of 3.279 is also high, indicating that the model’s predictions have a relatively large deviation from the true values. The MAE of 2.613 is lower than the RMSE, but still relatively high. The R^2^ score of 0.855 suggests that the model explains 85.5% of the variation in the data, which is a decent performance. For the Decision Tree model, the MSE is much lower at 1.766, indicating that the model’s predictions have a smaller average squared difference from the true values. The RMSE of 1.329 is also lower, suggesting that the model’s predictions have a smaller deviation from the true values. The MAE of 0.931 is the lowest of all the models, indicating that the model’s predictions have a smaller average absolute difference from the true values. The R^2^ score of 0.976 suggests that the model explains 97.6% of the variation in the data, which is a very good performance. For the Random Forest model, the MSE of 3.761 is higher than the Decision Tree model, but lower than the SVM model. The RMSE of 1.939 is between the two other models. The MAE of 1.408 is also between the two other models. The R^2^ score of 0.949 is lower than the Decision Tree model, but still a good performance. For the AdaBoost model, the MSE of 0.022 is the lowest of all the models, indicating that the model’s predictions have the smallest average squared difference from the true values. The RMSE of 0.147 is also the lowest of all the models, suggesting that the model’s predictions have the smallest deviation from the true values. The MAE of 0.022 is the same as the MSE, indicating that the model’s predictions have the smallest average absolute difference from the true values. The R^2^ score of 1.000 suggests that the model explains 100% of the variation in the data, which is a perfect performance.

Table 3 displays the performance metrics of four different machine learning models for predicting a given dataset. The metrics include Mean Squared Error (MSE), Root Mean Squared Error (RMSE), Mean Absolute Error (MAE), and R-squared (R^2^). The SVM model had the highest MSE, RMSE, and MAE, indicating relatively poor performance. The Decision Tree model had the lowest MSE, RMSE, and MAE, and the highest R^2^ score, indicating the best performance among the models. The Random Forest and AdaBoost models had intermediate performance metrics, with the AdaBoost model achieving the lowest MSE, RMSE, and MAE, and the highest R^2^ score, indicating the best overall performance among the models. Overall, the results suggest that the AdaBoost model may be the most effective for predicting the given dataset.

### Feature correlations and feature selection

In our study, we also examined the correlations between the features used in our machine learning models and their importance in predicting the Lumbar Angle and Pelvic Tilt measurements. Understanding these correlations and feature importance can provide insights into the underlying factors that influence these measurements and can guide the development of effective interventions.

To evaluate the feature correlations, we calculated the Pearson correlation coefficient between each feature and the Lumbar Angle and Pelvic Tilt measurements. The results of our analysis showed that some features had a strong positive or negative correlation with the Lumbar Angle and Pelvic Tilt measurements, while others had a weaker correlation. Our analysis revealed that some features were more important than others in predicting these measurements. Overall, our analysis of the feature correlations and feature importance provides valuable insights into the factors that influence the Lumbar Angle and Pelvic Tilt measurements. These findings can guide the development of interventions that target these factors to improve outcomes related to sexual function, urinary incontinence, quality of life, physical performance, and muscle strength in females.

To determine the most features that is more correlated to the prediction of Pelvic tilt and Lumbar angle for each class (Normal/Abnormal), we use the Pearson’s correlation method. Table 4 shows the Pearson correlations of the all features.

**Table 4:** Pearson’s correlation of the features.

First feature	Second feature	Correlation	First feature	Second feature	Correlation
VAS	UDI	0.91	Thickness	UDI	− 0.66
Pelvic tilt	VAS	0.87	Thickness	pelvic tilt	− 0.68
Force	Oxford	0.85	Thickness	Mobility	− 0.69
Lumbar angle	UDI	0.84	Thickness	VAS	− 0.7
Oxford	FSFI	0.82	OSW	FSFI	− 0.72
Pelvic tilt	UDI	0.81	Force	pelvic tilt	− 0.8
Mobility	VAS	0.79	Force	lumbar angle	− 0.82
Lumbar angle	pelvic tilt	0.78	pelvic tilt	Oxford	− 0.82
Force	FSFI	0.78	UDI	FSFI	− 0.82
Pelvic tilt	Mobility	0.76	VAS	FSFI	− 0.86
Pelvic tilt	OSW	0.74	Force	VAS	− 0.87
Thickness	Oxford	0.73	UDI	Oxford	− 0.87
Lumbar angle	Mobility	0.73	VAS	Oxford	− 0.89

Feature selection is a process used in machine learning to identify the most relevant and useful features from a set of features that are used to train a model. The goal of feature selection is to improve the accuracy and efficiency of the model by reducing the number of features used for training.

In the table provided, different feature selection techniques were used to determine the most important features in predicting the outcome measures related to sexual function, quality of life, physical performance, and muscle strength in females. The techniques used include F-value selector, mutual information selector, RFE with logistic regression, Select from model with random forests, and variance thresholding.

The most important features identified by each technique varied, but some features were consistently identified across multiple techniques. For example, lumbar angle, pelvic tilt, VAS, UDI, and Oxford scale were identified as important features by at least two of the techniques. This suggests that these features may have a significant impact on the outcome measures related to sexual function, quality of life, physical performance, and muscle strength in females. Overall, the use of feature selection techniques can help to improve the accuracy and efficiency of machine learning models, and can provide valuable insights into the most important features that contribute to the outcome measures of interest. These insights can guide the development of interventions that target these features to improve outcomes in females. Table 5 shows the feature selection techniques and the most important features.

**Table 5:** Feature selection techniques and the most important features.

Technique	Most important features
F-value selector	([‘force’, ‘lumbar angle’, ‘VAS’, ‘UDI’, ‘oxford’])
Mutual information selector	([‘pelvic tilt’, ‘VAS’, ‘UDI’, ‘oxford’, ‘FSFI’])
RFE with logistic regression	([‘lumbar angle’, ‘mobility’, ‘OSW’, ‘UDI’, ‘FSFI’])
Select from model with random forests	([‘lumbar angle’, ‘pelvic tilt’, ‘VAS’, ‘UDI’, ‘oxford’])
Variance thereholding	([‘BMI’, ‘force’, ‘lumbar angle’, ‘pelvic tilt’, ‘mobility’, ‘VAS’, ‘OSW’, ‘UDI’, ‘oxford’, ‘FSFI’’, )

For our study, several well-established techniques were applied to identify the most predictive variables:

- Pearson’s correlation analysis.
- F-value selector ranked features based on ANOVA F-statistics to highlight significant predictors of pelvic tilt.
- Mutual information and recursive feature elimination (RFE) with logistic regression further reduced dimensionality.
- Random forest models internally evaluated feature importance through Gini impurity/information gain criteria.
- Variance thresholding removed low-variance features unlikely to impact predictions.

The criteria we used to evaluate importance were:

- Statistical significance based on *p*-values and F-statistics.
- Information gain/Gini importance scores from tree-based models.
- Stability of selection across different techniques.

By employing multiple filter and wrapper methods, we aimed to identify a robust set of top predictive variables for our problem in an interpretable manner.

Based on the feature selection results, we recommended the Random Forests technique as it identified the most important features.

Some key reasons:

- Random Forests internally evaluates features based on their contribution to prediction, without being influenced by correlation among features like F-value selection.
- It selected features consistently identified as important by other methods like pelvic tilt, lumbar angle, VAS, UDI, Oxford scale.
- Features like pelvic tilt, lumbar angle are directly relevant to predicting changes, aligned with our goal.
- Variance thresholding retained too many features without distinguishing most predictive ones.
- While other methods like RFE + LR identified marginally different features, RF agreed with majority.
- RF is a highly versatile and accurate machine learning approach suitable for this type of medical data.

Random Forests’ feature importance criterion and ability to capture interactions/nonlinear effects makes it best suited for this dataset/problem. Identifying core predictive features is important for model interpretability and validity.

## Discussion and future work

Urinary incontinence (UI) is a prevalent condition characterized by uncontrolled urine leakage. It is associated with pelvic floor dysfunction, which involves the impairment of pelvic floor muscles (PFM) and can compromise trunk and lumbo-pelvic stability. Assessing the alignment and posture of the spine in the lower back region and pelvis is crucial in evaluating pelvic floor dysfunction, as these factors are directly related to female pelvic floor dysfunction. The traditional methods of assessing pelvic tilt and lumbar angle, which are important parameters in evaluating pelvic floor dysfunction, involve manual measurements. However, these methods are time-consuming and subject to variability, which can limit their accuracy and reliability. Therefore, there is a need for alternative approaches that are faster, more accurate, and less prone to variability.

In this study, we aimed to predict the activity of various core muscles in multiparous women with pelvic floor dysfunction using multiple scales, instead of relying on ultrasound imaging. We employed machine learning techniques, including decision tree, support vector machine (SVM), random forest, and AdaBoost models, to predict pelvic tilt and lumbar angle using a training set. We then evaluated the performance of these models on a test set using metrics such as mean squared error (MSE), root mean squared error (RMSE), mean absolute error (MAE), and coefficient of determination (R^2^). The results of our study demonstrated promising outcomes in predicting pelvic tilt and lumbar angle. The prediction of pelvic tilt achieved R^2^ values greater than 0.9, with the AdaBoost model performing best with an R^2^ value of 0.944. The prediction of lumbar angle yielded slightly lower results, with the decision tree model achieving the highest R^2^ value of 0.976.

Developing a machine learning model to predict pelvic tilt and lumbar angle has the potential to revolutionize the assessment and management of pelvic floor dysfunction. By providing faster, more accurate, and more objective assessments compared to traditional manual methods, this approach can enhance the efficiency and effectiveness of rehabilitation programs for pelvic floor dysfunction in physical therapy. Moreover, it can contribute to improving the quality of life for women affected by urinary incontinence worldwide.

However, it is important to acknowledge the limitations of our study. The generalizability of the results may be influenced by the specific characteristics of the study population, as well as the chosen machine learning models and scales used. Further research is needed to validate the findings in larger and more diverse populations, as well as to explore the potential application of these models in clinical settings. Additionally, the integration of other relevant variables and the comparison of machine learning models with existing assessment methods would be valuable avenues for future investigation.

The accumulating successes of ML have stimulated interest across medical disciplines seeking new solutions. By leveraging datasets, ML techniques may reveal correlations to aid conditions traditionally viewed as difficult to predict, such as outcomes following certain spine treatments. Continued progress depends on assembling representative patient information to develop robust algorithms applicable to real-world clinical scenarios. This study explores the capability of ML methods for a spine-related application^42,43^.

This is study put forward the following findings:

1. Showed a greater positive correlation between lumbar angle’, ‘pelvic tilt’, ‘VAS’, ‘UDI’, ‘oxford’.
  Our findings also show a correlation between elevated urogenital discomfort in women and elevated Low back pain (LBP) intensity, disability, lumbar angle, and pelvic tilt. Additionally, decreased VAS, FSFI, pelvic tilt, and lumbar angle were associated with increased PFM force.
2. The result is supported with this study, found that increasing in the sagittal spinal curvatures, pelvic tilt, and Lumbopelvic mobility was seen in women with UI compared to women without UI, in this study. Most of the women with UI had LBP. The urogenital distress was related to LBP and disability. It was concluded that sagittal spinal alignment and lumbopelvic hypermobility should be taken into consideration in the treatment of UI^44^.
3. One possible explanation for this phenomenon is that proper spinal alignment and normal curvatures can provide protection to the pelvis and pelvic floor against direct intra-abdominal forces, while also facilitating efficient contraction of the pelvic floor muscles (PFMs). Biomechanically, the positioning of all parts of the spine and pelvis are interconnected. Therefore, changes in the lumbar lordosis may be caused by postural adjustments in either the pelvis or the thoracic spine^45^. Moreover, the angle of the sacrum is related to the degree of lumbar lordosis, and the degree of lumbar lordosis is related to the degree of pelvic tilt^46^. A cadaveric study by Pool-Goudzwaard et al.^44^ showed that simulated tension of the pelvic floor muscles (PFMs) resulted in a significant 8.5% increase in stiffness of the sacroiliac joints and a backward rotation of the sacrum. The authors proposed that heightened PFM activity could enhance pelvic stability and improve the transfer of load through the lumbopelvic region.
4. Our study explored the potential of machine learning algorithms in predicting Lumbar Angle and Pelvic Tilt measurements in females. The findings demonstrated that machine learning models can effectively and precisely forecast these measurements, indicating their potential clinical relevance in managing conditions such as sexual function, quality of life, physical performance, and muscle strength in females.
5. The use of machine learning algorithms can provide several advantages over traditional methods of predicting Lumbar Angle and Pelvic Tilt measurements. Machine learning models can handle large amounts of complex data and can identify patterns and relationships that may not be apparent through traditional statistical methods. Additionally, machine learning models can be trained on large datasets, which can improve their accuracy and reliability.
  Our study used several different machine learning algorithms, including Decision Tree, SVM, Random Forest, and AdaBoost regressions. The results of our study showed that the Decision Tree and AdaBoost models achieved the best performance in predicting the Pelvic Tilt measurements, while the Random Forest and AdaBoost models achieved the best performance in predicting the Lumbar Angle measurements.
6. In addition to predicting Lumbar Angle and Pelvic Tilt measurements, our study also investigated the correlations between several features and these measurements. Our analysis revealed that some features had a strong correlation with Lumbar Angle and Pelvic Tilt measurements, while others had a weaker correlation.
7. Our study also used several feature selection techniques to identify the most important features in predicting the outcome measures related to sexual function, quality of life, physical performance, and muscle strength in females. The results of our study showed that lumbar angle, pelvic tilt, VAS, UDI, and Oxford scale were consistently identified as important features across multiple techniques.
8. Developing an effective user interface is indeed crucial but was beyond the scope of our current study.

As this was a proof-of-concept study focused on validating the technical feasibility of our predictive modeling approach, we have not yet implemented a full clinical decision support interface.

However, designing an intuitive interface tailored to healthcare practitioners’ needs a priority in future work. Some aspects we plan to address include:

- Conducting user interviews/focus groups with clinicians to understand essential design requirements.
- Prototyping mobile/web apps with easy predict/report functions integrated into workflow.
- Incorporating clinical decision aids based on predicted changes and established guidelines.
- Implementing interactive education/visualization of model internals for transparency.
- Pilot testing prototypes for usability, usefulness and real-world performance.
- Gathering clinician feedback to iteratively refine the interface design.

While the current models demonstrated predictive performance, interpretability for clinicians and patients was not sufficiently addressed. Some ways we plan to enhance interpretability in future work include:

- Developing visual and textual explanations of model predictions to demonstrate how different factors contribute to results. This can leverage techniques like SHAP values and LIME.
- Gradually explaining model components to non-technical audiences, starting with easily understood concepts like decision trees before introducing more complex algorithms.
- Validating that explanations are clear and actionable for intended end-users through pilot testing, surveys and interviews.
- Integrating explanations directly into the clinical decision support interface to provide context alongside predictions.
- Illustrating model limitations and uncertainties to manage expectations and encourage responsible, augmented decision making.

## Limitations

- One of the strengths of this project is the dataset of pelvic tilt and lumbar angle measurements that were gathered. This dataset allowed for a comprehensive analysis of the factors that contribute to changes in pelvic tilt and lumbar angle in cases of incontinence and sexual dysfunction. Additionally, the use of cross-validation techniques helped to ensure that the models were robust and not overfit to the training data.
- There are also limitations to this project, such as the potential for selection bias in the gathered dataset, and the need for further validation and testing in clinical settings. However, these limitations can be addressed in future studies.
- Overall, this project has demonstrated the potential for machine learning algorithms to be used in the diagnosis and treatment of incontinence and sexual dysfunction by accurately predicting changes in pelvic tilt and lumbar angle. This technology has the potential to revolutionize the way that healthcare professionals approach these conditions, leading to more effective and personalized treatment plans for affected women.

## Conclusions

The proposed framework underscores the importance of predicting lumbar angle and pelvic tilt in females with urinary incontinence (UI) and sexual dysfunction, aiming to support therapists in making informed therapeutic decisions. The study findings suggest that machine learning techniques can have a significant impact on physician decision-making regarding the selection of appropriate treatment methods. This research has the potential to contribute to the development of treatment guidelines for UI and sexual dysfunction patients, particularly those experiencing lower back pain (LBP) in conjunction with UI. The present paper successfully developed a machine learning algorithm capable of accurately predicting changes in pelvic tilt and lumbar angle in cases of urinary incontinence and sexual dysfunction. The application of this algorithm has the potential to enhance the diagnosis and treatment of these conditions, ultimately improving the quality of life for affected women. Among the evaluated models, the Decision Tree and AdaBoost models exhibited the best performance across all four metrics for predicting pelvic tilts and lumbar angles. However, the SVM and Random Forest models displayed certain strengths in specific metrics while demonstrating lower performance in others. The choice of which model to utilize would depend on the specific requirements of the problem at hand and the relative importance of the different metrics for the given task. Careful consideration should be given to selecting the most appropriate model based on the desired outcome.

## Data Availability

The dataset used in this study is public and all test data are available at: (https://github.com/tarekhemdan/Lumber-Angle---Pelvic-Tilt-Prediction/blob/main/Data_Fix_Final.csv). Mirror 1: https://shorturl.at/mrxJK, Mirror 2: https://rb.gy/a2r00.

## References

[1] Scutelnic G, Gutu C (2023). Incontinence of urine in women. Diagnosis and treatment. Sci. Collect. InterConf.

[2] Peate I (2019). Urinary incontinence in women: treatment recommendations. Br. J. Nurs..

[3] Doumouchtsis SK (2023). An International Continence Society (ICS)/ International Urogynecological Association (IUGA) joint report on the terminology for the assessment and management of obstetric pelvic floor disorders. Int. Urogynecol. J..

[4] Âmiri M, Mohseni Bandpei MA, Rahmani N (2010). A comparison of pelvic floor muscle endurance and strength between patients with chronic low back pain and healthy subjects. J. Mazandaran Univ. Med. Sci..

[5] Çelenay Ş, Kaya D (2017). Relationship of spinal curvature, mobility, and low back pain in womenwith and without urinary incontinence. Turk. J. Med. Sci..

[6] Smith MD, Coppieters MW, Hodges PW (2007). Postural response of the pelvic floor and abdominal muscles in women with and without incontinence. Neurourol. Urodyn..

[7] Prouza, A. & Hashim, H. Mesh complications and their management. *Textb. Female Urol. Urogynecol. Surg. Perspect.* 868 (2023).

[8] Le Huec JC, Aunoble S, Philippe L, Nicolas P (2011). Pelvic parameters: origin and significance. Eur. Spine J..

[9] Walker ML, Rothstein JM, Finucane SD, Lamb RL (1987). Relationships between lumbar lordosis, pelvic tilt, and abdominal muscle performance. Phys. Ther..

[10] Szolovits P (2019). Artificial Intelligence in Medicine.

[11] Suzuki H, Inaba Y, Kobayashi N, Ishida T, Ike H, Saito T (2016). Postural and chronological change in pelvic tilt 5 years after total hip arthroplasty in patients with developmental dysplasia of the hip: A three-dimensional analysis. J. Arthroplasty.

[12] Lembeck B, Mueller O, Reize P, Wuelker N (2005). Pelvic tilt makes acetabular cup navigation inaccurate. Acta Orthop..

[13] Nishihara S, Sugano N, Nishii T, Ohzono K, Yoshikawa H (2003). Measurements of pelvic flexion angle using three-dimensional computed tomography. Clin. Orthop. Relat. Res..

[14] Gulshan V (2016). Development and validation of a deep learning algorithm for detection of diabetic retinopathy in retinal fundus photographs. JAMA.

[15] Kawakami E (2019). Application of artificial intelligence for preoperative diagnostic and prognostic prediction in epithelial ovarian cancer based on blood biomarkers. Clin. Cancer Res..

[16] Esteva A (2017). Dermatologist-level classification of skin cancer with deep neural networks. Nature.

[17] Beam AL, Manrai AK, Ghassemi M (2020). Challenges to the reproducibility of machine learning models in health Care. JAMA.

[18] Babisch JW, Layher F, Amiot L-P (2008). The rationale for tilt-adjusted acetabular cup navigation. JBJS.

[19] Lazennec JY (2003). Hip-spine relationship: A radio-anatomical study for optimization in acetabular cup positioning. Surg. Radiol. Anat. SRA.

[20] Schwartz JT (2021). Deep learning automates measurement of spinopelvic parameters on lateral lumbar radiographs. Spine.

[21] Shieh G, Jan S, Randles R (2006). On power and sample size determinations for the Wilcoxon–Mann–Whitney test. J. Nonparametr. Stat..

[22] Mørkved S, Salvesen KÅ, Bø K, Eik-Nes S (2004). Pelvic floor muscle strength and thickness in continent and incontinent nulliparous pregnant women. Int. Urogynecol. J..

[23] Arab AM, Behbahani RB, Lorestani L, Azari A (2010). Assessment of pelvic floor muscle function in women with and without low back pain using transabdominal ultrasound. Man. Ther..

[24] Tosun OC (2016). Assessment of the effect of pelvic floor exercises on pelvic floor muscle strength using ultrasonography in patients with urinary incontinence: A prospective randomized controlled trial. J. Phys. Ther. Sci..

[25] Thompson JA, O’sullivan PB, Briffa NK, Neumann P (2006). Assessment of voluntary pelvic floor muscle contraction in continent and incontinent women using transperineal ultrasound, manual muscle testing and vaginal squeeze pressure measurements. Int. Urogynecol. J..

[26] Skorupska K, Grzybowska ME, Kubik-Komar A, Rechberger T, Miotla P (2021). Identification of the Urogenital Distress Inventory-6 and the Incontinence Impact Questionnaire-7 cutoff scores in urinary incontinent women. Health Qual. Life Outcomes.

[27] Chen J, Ren Y, Zhu L (2020). Correlation between modified Oxford grading scale and pelvic floor surface electromyography in assessment of pelvic floor muscle function in female patients with stress urinary incontinence. Zhonghua Yi Xue Za Zhi.

[28] Lai J (2023). Treatment of degenerative lumbar scoliosis using transforaminal lumbar interbody fusion based on the concept of intervertebral correction. Int. Orthop..

[29] Aparicio VA, Marín-Jiménez N, Flor-Alemany M, Acosta-Manzano P, Coll-Risco I, Baena-García L (2023). Effects of a concurrent exercise training program on low back and sciatic pain and pain disability in late pregnancy. Scand. J. Med. Sci. Sports.

[30] Summers RM (2018). Deep learning lends a hand to pediatric radiology. Radiology.

[31] Isidori AM (2010). original research—outcomes assessment: Development and validation of a 6-item version of the female sexual function index (FSFI) as a diagnostic tool for female sexual dysfunction. J. Sex. Med..

[32] Hastie T, Tibshirani R, Friedman J (2009). The Elements of Statistical Learning.

[33] Breiman L (2001). Random forests. Mach. Learn..

[34] Rédei GP (2008). Encyclopedia of Genetics, Genomics, Proteomics and Informatics.

[35] “Support Vector Machines (SVM) | SpringerLink.” Accessed: 25 Mar 2023. [Online]. Available: https://link.springer.com/chapter/10.1007/978-3-319-67371-4_10

[36] Arefinia A, Bozorg-Haddad O, Zolghadr-Asli B (2022). Using support vector machine (SVM) in modeling water resources systems. Computational Intelligence for Water and Environmental Sciences, Studies in Computational Intelligence.

[37] Kufel J (2023). What is machine learning, artificial neural networks and deep learning?—Examples of practical applications in medicine. Diagnostics.

[38] Burduk R, Choraś M, Choraś RS (2018). The AdaBoost algorithm with linear modification of the weights. Image Processing and Communications Challenges, Advances in Intelligent Systems and Computing.

[39] Strojek K, Strączyńska A, Radzimińska A, Weber-Rajek M (2023). The effects of extracorporeal magnetic innervation in the treatment of women with urinary incontinence: A systematic review. J. Clin. Med..

[40] Tosun OC, Keser I, Dayican DK, Yavuz O, Tosun G, Kurt S (2023). Does multiple-component intensive pelvic floor muscle training decrease muscle fatigue and symptoms in women with urinary incontinence?. Int. Urogynecol. J..

[41] Parnianpour M, Davoodi M, Forman M, Rose DJ (1994). The normative database for the quantitative trunk performance of female dancers: Isometric and dynamic trunk strength and endurance. Med. Probl. Perform. Art..

[42] Joshi RS, Haddad AF, Lau D, Ames CP (2019). Artificial intelligence for adult spinal deformity. Neurospine.

[43] Schwartz JT, Gao M, Geng EA, Mody KS, Mikhail CM, Cho SK (2019). Applications of machine learning using electronic medical records in spine surgery. Neurospine.

[44] Pool-Goudzwaard A, van Dijke GH, van Gurp M, Mulder P, Snijders C, Stoeckart R (2004). Contribution of pelvic floor muscles to stiffness of the pelvic ring. Clin. Biomech..

[45] Nakipoğlu GF, Karagöz A, Ozgirgin N (2008). The biomechanics of the lumbosacral region in acute and chronic low back pain patients. Pain Physician.

[46] Levine D, Whittle MW (1996). The effects of pelvic movement on lumbar lordosis in the standing position. J. Orthop. Sports Phys. Ther..

